# Structure-Guided Designing and Evaluation of Peptides Targeting Bacterial Transcription

**DOI:** 10.3389/fbioe.2020.00797

**Published:** 2020-09-08

**Authors:** Gundeep Kaur, Srajan Kapoor, Soni Kaundal, Dipak Dutta, Krishan Gopal Thakur

**Affiliations:** ^1^Structural Biology Laboratory, G. N. Ramachandran Protein Centre, Council of Scientific and Industrial Research-Institute of Microbial Technology, Chandigarh, India; ^2^Molecular Microbiology Laboratory, Council of Scientific and Industrial Research-Institute of Microbial Technology, Chandigarh, India

**Keywords:** RNA polymerase, CarD, peptide-based inhibitors, *Mycobacterium tuberculosis*, bacterial transcription, protein-protein interaction

## Abstract

The mycobacterial RNA polymerase (RNAP) is an essential and validated drug target for developing antibacterial drugs. The β-subunit of *Mycobacterium tuberculosis (Mtb)* RNAP (RpoB) interacts with an essential and global transcription factor, CarD, and confers antibiotic and oxidative stress resistance to *Mtb*. Compromising the RpoB/CarD interactions results in the killing of mycobacteria, hence disrupting the RpoB/CarD interaction has been proposed as a novel strategy for the development of anti-tubercular drugs. Here, we describe the first approach to rationally design and test the efficacy of the peptide-based inhibitors which specifically target the conserved PPI interface between the bacterial RNAP β/transcription factor complex. We performed *in silico* protein-peptide docking studies along with biochemical assays to characterize the novel peptide-based inhibitors. Our results suggest that the top ranked peptides are highly stable, soluble in aqueous buffer, and capable of inhibiting transcription with IC_50_ > 50 μM concentration. Using peptide-based molecules, our study provides the first piece of evidence to target the conserved RNAP β/transcription factor interface for designing new inhibitors. Our results may hence form the basis to further improve the potential of these novel peptides in modulating bacterial gene expression, thus inhibiting bacterial growth and combating bacterial infections.

## Graphical Abstract

Bacterial RNAP interacts with transcription factors, such as CarD and TRCF, which stabilizes the transcription initiation complex and activates transcription. Interface peptide-based inhibitors target and interact with RNAP (β and interfere with RNAP/transcription factor interaction, and thus affect the bacterial transcription.

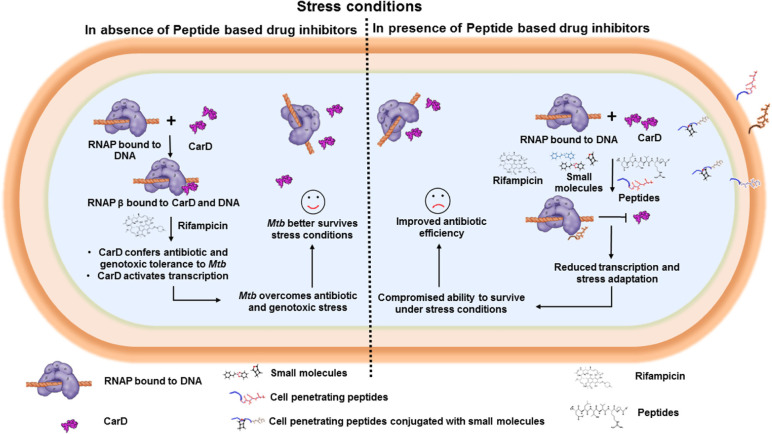

## Introduction

Tuberculosis (TB) remains a major cause of death in the world (WHO, 2016). Rifampicin, the lynchpin for TB, targets bacterial RNA Polymerase (RNAP) (Mcclure and Cech, 1978; Campbell et al., 2001), a key and essential enzyme involved in transcription. RNAP is conserved in structure and function in all bacteria (Werner, 2008; Werner and Grohmann, 2011). Bacterial RNAP is a proven and validated target for developing antibacterial drugs (Chopra, 2007; Ho et al., 2009; Ma et al., 2016). But due to the emergence of the multi-drug and extremely drug-resistant strains of pathogenic bacteria (Lushniak, 2014; Alos, 2015; Ventola, 2015a), the treatment of infectious diseases is becoming challenging. Thus, there is an urgent need to develop novel drug molecules against newer targets or newer sites in the established targets in bacteria (Gould, 2010; Mendelson, 2015; Ventola, 2015b). So, the potent novel antibacterial drugs should ideally target bacterial RNAP in such a way that they do not show cross-resistance with already approved drugs, such as rifampicin.

Bacterial RNAP is a multi-subunit complex comprised of five subunits (α_2_ββ′ω) which associate reversibly with an σ-factor to form a holoenzyme (Helmann and Chamberlin, 1988). Different subunits of RNAP interact with several transcription factors, like CarD (Stallings et al., 2009), WhiB7 (Burian et al., 2013), UvrD helicase (Epshtein et al., 2014), RNA polymerase binding protein A (RbpA) (Newell et al., 2006), Transcription Repair Coupling Factor (TRCF) (Selby and Sancar, 1995a, b), Rho (Das et al., 1978), etc., at various stages of transcription. Some of the transcription regulators, such as CarD (Weiss et al., 2012) and TRCF (Westblade et al., 2010), interact exclusively with β-subunit of RNAP and modulate transcription. *Mtb* and *Escherichia coli* RNAP differ in their abilities to form an open promoter complex (Rammohan et al., 2015, 2016). *Mtb* RNAP, being a relatively slow and unstable enzyme, requires the association with the essential accessory factors like CarD and RbpA (Hu et al., 2012; Davis et al., 2015) to increase the rate of transcript production *in vitro* and stabilize the unstable RNAP open complex (Davis et al., 2015) by preventing transcription bubble collapse (Rammohan et al., 2015).

CarD has been proposed as an attractive drug target, as *Mycobacterium smegmatis* lacking CarD has been shown to be susceptible to killing by Reactive Oxygen Species (ROS), ciprofloxacin, and starvation (Stallings et al., 2009). Although absent in *E. coli* and eukaryotes, CarD is widely present across actinomycetes. Weakening the interaction between RNAP and CarD makes the mycobacteria susceptible to antibiotic and oxidative stress, thereby killing the bug (Weiss et al., 2012). Recent structural studies on *Mtb* CarD and its homologs (Gallego-Garcia et al., 2012, 2014; Gulten and Sacchettini, 2013; Srivastava et al., 2013; Kaur et al., 2014; Bernal-Bernal et al., 2015; Kaur et al., 2018) suggests that CarD^NTD^ (N-Terminal Domain of CarD), also known as RNAP Interacting Domain (RID), adopts a Tudor-like fold, similar to *E. coli and Tth* TRCF^NTD^ (N-Terminal Domain of transcription-repair coupling factor, involved in coupling transcription to DNA repair)(Westblade et al., 2010). Both CarD^NTD^ and TRCF^NTD^ interact with β1 β2 domain (RpoBtr) and β1 domain of β-subunit of RNAP, respectively (PDBID’s: 4KBM and 3MLQ) (Westblade et al., 2010; Weiss et al., 2012).

Targeting and inhibiting PPIs using peptide-based inhibitors is an attractive strategy to block a number of important targets involved in pathological conditions (Fry, 2015; Bakail and Ochsenbein, 2016). However, it is quite challenging to design the peptide-based inhibitors. It is also difficult to characterize the protein-peptide interactions since the interface peptides are quite flexible and may adopt multiple conformations in solution (Pelay-Gimeno et al., 2015; Lamiable et al., 2016). Nonetheless, the market for peptide-based drugs is expanding day-by-day (Craik et al., 2013; Uhlig et al., 2014) and they are emerging as attractive candidates for disrupting PPI (Fosgerau and Hoffmann, 2015). Recently, Shanmuga Priya et al. (2018), reported a virtual screening based strategy to identify and target small molecules against CarD.

In this study, we describe the first approach to rationally design and test the efficacy of the peptide-based inhibitors which specifically target the conserved RpoBtr/transcription factor PPI interface (Supplementary Figure 1). We hypothesized that binding of the peptide-based inhibitors to the solvent exposed protein interface of *Mtb* RpoBtr might interfere with the binding of an essential transcription factor, CarD, consequently affecting the stability of the open promoter complex and reducing the amount of transcript formed. Our results suggest that one of the rationally designed peptide-based inhibitors was able to reduce the CarD mediated transcription activation, hence demonstrating the potential of targeting this conserved PPI interface for developing improved molecules to combat mycobacterial infections in the future.

## Materials and Methods

### Identification of the Novel Druggable Site in Bacterial RNAP β and Structure-Guided Designing of Novel Synthetic Interface Peptides

Four different peptides were designed manually after analyzing the structures of RNAP β/CarD (PDBID: 4KBM) and *Tth* TRCF (PDBID: 3MLQ). Taking into account the residues involved in the interaction between *Mtb* RNAP β/CarD and *Tth* RNAP/TRCF, the interface peptide approach was used to design the peptides (7–8 amino acids in length), which could target and bind bacterial RNAP (Table 1).

**TABLE 1:** The details of the designed interface peptides and the parameters obtained after modeling peptides using PEP-FOLD3.1 web server (Lamiable et al., 2016).

Peptide ID	Selected protein residues	Peptide sequence	MW_exp_ (Da)	Clusters generated	OPEP score
1	TRCF Glu358-Glu366	EGKLYPVE	933.47	34	−4.85
2	RpoB Thr138-Thr145	TGEIKSQT	862.43	18	−4.66
3	CarD Asp43-Pro49	DLTVRVP	798.46	14	−3.48
4	RbpA Gly37-Pro43	GEEFEVP	805.35	7	−2.55

### Peptide Synthesis and Peptide Modeling

The designed peptides were commercially synthesized using solid phase peptide synthesis strategy and purchased from GL Biosciences, China. The 3D structures of the designed peptides were predicted and modeled using the PEP-FOLD3.1 web server (Lamiable et al., 2016). PEP-FOLD 3.1 predicts the structure of peptides on the basis of Hidden Markov model conformations.

### LC-MS Analysis of Peptides

The stability of all the designed peptides was analyzed using Liquid chromatography-mass spectrometry (LC-MS) using Agilent Technologies (model G6550A). Peptide 1, 2, and 4 were injected in the C18 column equilibrated in a negative mode at a flow rate of 0.5 mL/min using water as a mobile phase. Peptide 3 was injected in the C18 column equilibrated in a positive mode at a flow rate of 0.5 mL/min using water as a mobile phase.

### Molecular Docking Analyses of Designed Peptides and RpoB

The crystal structure of the β1β2 domain of β-subunit of RNAP (RpoBtr) in complex with CarD (PDBID 4KBM) was retrieved from Protein Data Bank^1^ and pre-processed using the Protein Preparation wizard 2.2 of the Maestro, Schrödinger software suite (Schrödinger, 2017). The CarD was removed at the protein preparation stage in order to prepare RpoBtr as the receptor. H-atoms were added and the bond orders were assigned. This was followed by the creation of zero-order bonds to metals and disulfide bonds. Missing side chains of the RpoBtr were modeled and the loops were refined using Prime 3.0 (Jacobson et al., 2002; Jacobson et al., 2004) module of Schrödinger. Water molecules were deleted, and the ionization and tautomeric states in the pH range 7 ± 2 were generated by Epik (Shelley et al., 2007). The H-bonds were assigned and optimized by selecting PROPKA at pH 7. The structure was finally restrained and minimized using OPLS3 force field (Harder et al., 2016). All the peptides were prepared using LigPrep (Schrödinger, 2017) module of Schrödinger for generating all possible energetically minimum conformers. Molecular docking was initiated by defining the prepared RNAP β as a receptor and providing the binding site information required to dock the peptides on the receptor followed by the generation of the grid file. Thereafter, the generated grid file was used for molecular docking using the SP-peptide module (Friesner et al., 2004) of Maestro. The sampling of the peptides was kept flexible throughout the process of docking. The docking results were analyzed for the best-docked pose for each peptide-RNAP β on the basis of Glide SP score. All the molecular graphics figures were prepared using Pymol software (Delano, 2002).

### Physiochemical Properties and Biological Assessment of Lead Peptides

The lead peptide-based inhibitors were assessed for their physiochemical properties. The molecular weight, theoretical isoelectric point (pI), overall charge, aliphatic index value, instability index, and average hydropathy (GRAVY) were calculated using Protparam server (Wilkins et al., 1999). The subcellular localization of the lead peptide-based inhibitor was determined using the CELLO prediction server v.2.5 (Yu et al., 2004) which helps to predict subcellular localization in eukaryotes.

### Cloning, Over-Expression, and Purification of Recombinant Proteins

The cloning, over-expression, and purification of *Mtb* CarD was carried out as per the procedures mentioned in Kaur et al. (2014, 2018). *Mtb* RpoBtr was cloned in pNIC28-Bsa4 between *Nde*I and *Hin*dIII sites. The over-expression and purification of RpoBtr was carried out in a similar way as described for CarD (Kaur et al., 2014), except the buffer used for purification for both CarD and RpoB in this study was 25 mM Tris-HCl pH 7.5, 100 mM NaCl, and 2 mM β-Mercaptoethanol (Buffer A). The over-expression and purification of *Mtb* RNAP holoenzyme was carried out as per the reported procedures (Banerjee et al., 2014).

### *In vitro* Transcription Assays

*In vitro* transcription assays were performed as mentioned in Banerjee et al. (2014) with slight modifications. sinP3 promoter DNA was PCR amplified and further gel extracted using Thermo Fischer Gel extraction kit. Transcription initiation complex was incubated with varying concentrations of each peptide (10 μM − 1 mM) followed by incubation with CarD (1 μM). The reaction was initiated with the addition of NTP mixture (final concentration 250 mM ATP, GTP, UTP, and 50 mM CTP containing 0.5 mCi α^32^P-CTP) and allowed to proceed for 60 s. The transcription was terminated by the addition of 2X Formamide loading dye (80% formamide, 10 mM EDTA, 0.01% Bromophenol Blue, 0.01% Xylene Cyanol, and 0.1%SDS). The terminated reactions were heated at 95°C for 5 min and loaded on the 10% Urea denaturing gel at 25 W for 2 h. The images were scanned used Fujifilm Phoshoimager (FLA 5000). Densitometry analysis was performed using Image J software (Perez and Pascau, 2013). The data presented is the average of three independent experiments.

## Results

### Structural Analysis of the Conserved RNAP β Subunit/Transcription Factor Binding Interface

**FIGURE 1:**
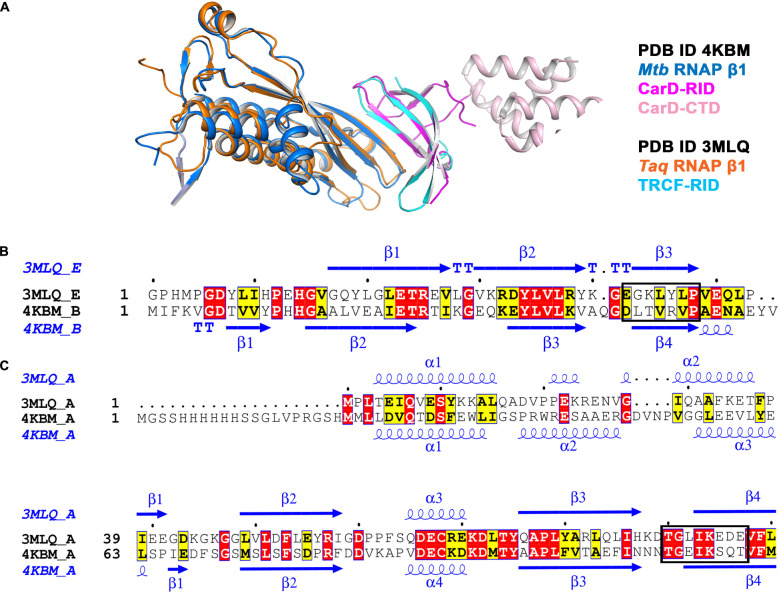
**(A)** Structural superposition of *Mtb* RNAP β1 (marine)/CARD-RID (magenta) [PDB ID 4KBM] with *Tth* RNAP β1 (orange)/TRCF-RID complex (cyan) [PDB ID 3MLQ] shows the solvent-exposed conserved protein-protein interface. **(B)** Structural and sequence alignment of *Mtb* CarD-RID (4KBM chain B) with *Tth* TRCF-RID (3MLQ chain E). *Mtb* CarD-RID with *Tth* TRCF-RID do not share significant sequence similarity but do share the same site for interaction with RNAP β1 (shown in the rectangular box). The figure was generated using ESPript 3 server (Robert and Gouet, 2014). **(C)** Structural and sequence alignment of *Mtb* RNAP β1β2 (4KBM chain A) with *Tth* RNAP β1 suggests that both *Mtb* RNAP β1β2 and *Tth* RNAP β1 (3MLQ chain A) share the conserved site for interaction with transcription factor-RID (shown in the rectangular box). The figure was generated using ESPript 3 server (Robert and Gouet, 2014).

In order to analyze the conserved bacterial RNAP β/transcription factor binding interface, we retrieved and analyzed the available X-ray crystal structures of bacterial RNAP β and the transcription factor complexes from the Protein Data Bank (PDB). The structural analysis of *Mtb* RpoBtr/CarD complex (PDB ID 4KBM) reveals that CarD binds RpoBtr in 1:1 stoichiometry, burying a relatively small surface area of about 500 Å^2^ (Figure 1A). The PPI interface is stabilized by eight stranded antiparallel β-sheets, where four-stranded antiparallel β-sheet are from the CarD^NTD^ and the four-stranded antiparallel β-sheet from the RpoBtr and eight hydrogen bonds (Gulten and Sacchettini, 2013). The PPI analysis suggests the role of “β-strand addition,” especially β-stand augmentation (Remaut and Waksman, 2006), which involves the addition of β-strand in CarD to β-strand in RNAP β or vice versa, which ultimately results in the formation of a continuous β-sheet. Similarly, the structure analysis of *Tth* RNAP β1/TRCF-RID complex (PDB ID 3MLQ) reveals that TRCF also binds β1 domain of β-subunit of RNAP in 1:1 stoichiometry (Figure 1A). Though CarD and TRCF do not share any significant sequence similarities, their secondary structure segments are more conserved (Figure 1B). Structural superposition of TRCF-RID and CarD^NTD^ yields a root mean square deviation (RMSD) of 1.19 Å, thus suggesting that both the transcription regulators containing tudor-like domains share the same interface for interaction with RNAP β. In addition, the RMSD between C_α_ atoms of RNAP β and *Tth* RNAP β1 is 1.14 Å, thus suggesting the conserved site of interaction between RNAP β/transcription factor-RID. Since the interface residues in RNAP involved in PPI in *Tth* RNAP β1/TRCF-RID and *Mtb* RpoBtr/CarD complexes are structurally conserved in both gram-positive and gram-negative bacteria (Supplementary Table 1 and Figures 1B,C), and absent in eukaryotes, targeting and inhibiting these PPIs might help in developing novel broad-spectrum antibacterial inhibitors.

### Sequence-Based Analysis for the Conservation of the RNAP β/CarD Binding Interface

To analyze the conservation of the binding interface of RpoBtr and CarD, we retrieved the whole genome sequencing data of several clinical multi-drug resistant (MDR) and extensively drug resistant (XDR) *Mtb* strains and performed BLASTp (Mcginnis and Madden, 2004) analysis [selected *Mycobacterium tuberculosis* complex (taxid:77643) as organism in drop down menu, max target sequences were set to 10,000, and the rest of the parameters were set as default] for both RpoB and CarD. We analyzed sequences of CarD and RpoB in several MDR and XDR strains of *Mtb* and observed that the residues involved in the CarD/RpoB interaction are highly conserved (Supplementary Figure 2), thus suggesting that CarD/RNAP β interactions are crucial for the survival of the bug. This prompted us to design inhibitors targeting this highly conserved RpoBtr/CarD interface which can potentially inhibit growth of even MDR and XDR *Mtb* strains.

### Design, 3D-Structure Prediction, and Analyses of Peptide-Based RNAP β/Transcription Factor Inhibitors

As the interaction interface between RNAP β1β2 domain and the transcription factor (CarD^NTD^ and TRCF-RID) (Westblade et al., 2010; Gulten and Sacchettini, 2013) is relatively large, flat, solvent-exposed, and devoid of the binding pocket, it is challenging to design small molecule-based inhibitors. We therefore aimed to design the peptide-based RNAP β/transcription factor inhibitors by selecting the key residues involved in the interaction at the conserved PPI interface between RNAP β and transcription factor complex. The sequence and molecular weight of the designed peptides are mentioned in Table 1. PEP-FOLD3.1 (Lamiable et al., 2016), an online server, was used to predict the *de novo* structures of the designed peptides by running 100 simulations for each peptide. PEP-FOLD3.1 provides the information in the form of models which are generated by OPEP (Optimized Potential for Efficient structure Prediction) energy. The topmost model with the lowest OPEP score having minimum energy and maximum stability for each peptide was selected for further computational analysis. The detailed parameters derived from PEP-FOLD3.1 are summarized in Table 1. The designed peptides were 7–8 amino acids in length and were commercially synthesized. The peptides were analyzed for their stability and solubility. All the peptides were soluble in water as well as in aqueous buffer with a solubility of > 10 mM. The LC-MS analysis of all the designed peptides shows that the peptides were stable in water even when stored for over a year at 4°C as the experimentally observed molecular weight of all peptides matched well with their theoretically calculated molecular weights (Supplementary Figure 3).

### Identification of the Potential Lead Peptide-Based RNAP β Inhibitors Using Molecular Docking

**FIGURE 2:**
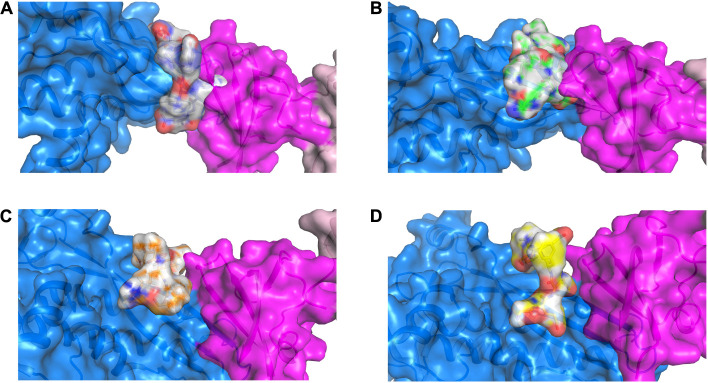
3D representation of the RNAP β (marine), CarD (magenta) and peptide-based inhibitor binding interface displaying the top ranked pose for each peptide (gray) when docked onto RNAP β. **(A)** RNAP β and peptide 1; **(B)** RNAP β and peptide 2; **(C)** RNAP β and peptide 3; and **(D)** RNAP β and peptide 4.

To examine the interaction between RpoBtr and the designed interface peptides, we performed molecular docking experiments where we docked the peptides on the RpoBtr (receptor) using the SP-peptide module of Glide in Maestro 9.8 of the Schrödinger software suite. The top ranking RpoBtr-docked peptide conformations were selected and analyzed for their glide score, docking score, and low binding energy values. The parameters obtained after molecular docking are listed in Table 2. All the protein-peptide complexes exhibited a multipose binding. The top scoring poses of the protein-peptide complexes were further analyzed manually (Figures 2A–D). Molecular docking results suggest that peptides 1 and 2 have a comparable binding affinity and docking scores and are relatively better than peptides 3 and 4. This is also in agreement with the interaction energy analysis performed using PEP-FOLD3.1 web server (Lamiable et al., 2016).

**TABLE 2:** Comparison of docking and binding energy scores (Kcal/mol) of potential shortlisted interface peptides targeting conserved RNAP β/transcription factor interface.

Peptide ID	Docking Score	Glide gScore	Van der Waals energy	Coulomb energy	Glide Emodel	Glide energy
1	–8.681	–8.795	–31.402	–26.689	–104.005	–58.091
2	–8.558	–8.574	–27.588	–26.531	–93.136	–54.123
3	–7.573	–7.944	–25.91	–27.19	–84.506	–53.124
4	–5.138	–5.382	–21.872	–11.086	–27.365	–32.598

The key residues involved in the interaction between RpoBtr-docked peptides (1–4) were determined using the 2D peptide interaction diagrams generated by Ligand Interaction script using Maestro (Schrödinger Inc.)^2^ (Supplementary Figures 4–7). The binding mode and the docking results suggest that the designed peptides 1 and 2 interact and bind at a similar binding site of RpoBtr/transcription factor, whereas the peptides 3 and 4 bind near the proximity of the binding site. Next, we examined the key residues involved in the interaction between RpoBtr-peptide 1 and RpoBtr-peptide 2 complexes. Eight intermolecular hydrogen bonds formed between RNAP β (Thr130, Glu132, Ser143, Thr145, Phe147, and Gln409) and peptide 1, which help in stabilizing the RpoBtr-peptide 1 complex. In addition, peptide 1 Asp1 forms a salt bridge with the RNAP β Glu132. Six intermolecular hydrogen bonds formed between RNAP β (Arg105, Asp107, Thr145, Gln409, Arg415) and peptide 2, which help in stabilizing the RpoBtr-peptide 2 complex. Furthermore, RNAP β Asp107 forms two salt bridges with peptide 2 Thr1 and Lys5. The surface representation showing the binding mode and the key residues involved in the interactions between RpoBtr and the peptides 1–4 are shown in Supplementary Figures 4–7.

### Physiochemical Properties and Biological Assessment of Lead Peptide

Protparam server (Wilkins et al., 1999) was used to assess the physiological properties of the lead RpoB/transcription factor complex peptide-based inhibitors. Peptides 1 and 2 have low molecular weights with an acidic nature and are highly stable with an instability index of 27.03 and 26.25, respectively. The aliphatic index values for both peptide 1 and 2 were relatively higher, suggesting these peptides are less hydrophobic. The subcellular localization in eukaryotes of all the designed peptides was predicted using the CELLO prediction server v.2.5 (Yu et al., 2004) which predicted the localization of all the peptides in mitochondrial and nuclear organelle with a high reliability score (2.044 and 1.057, respectively).

### *In vitro* Testing of the Peptide-Based RNAP β Inhibitors

**FIGURE 3:**
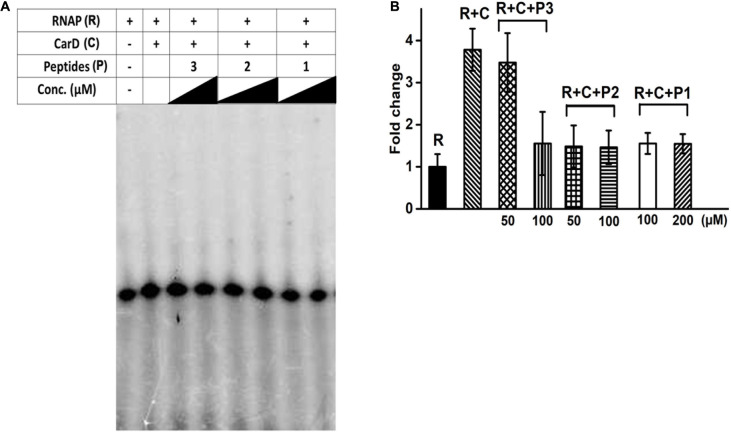
**(A)** *In vitro* transcription assay in the presence of varying concentrations of designed interface peptides (10 μM – 1 mM) and *Mtb* CarD. **(B)** Densitometry analysis of the transcripts obtained in the denaturing gel shown in **(A)** in the presence of peptides 1–3. Abbreviations: R, RNAP; C, CarD; P1, Peptide 1; P2, Peptide 2; P3, Peptide 3.

Our *in silico* docking experiments suggested that peptide-based inhibitors 1 and 2 have a higher affinity for bacterial RpoBtr as compared to other peptides. As reported earlier, CarD increases the rate of transcript production from sinP3 promoter (Srivastava et al., 2013). We hypothesized that the binding of peptide-based inhibitors to RpoBtr might compete with CarD for the binding site. As a result, transcription activation would be affected and only the basal level transcription will be observed. In order to test our hypothesis, we performed the *in vitro* transcription assays. In agreement with previously published studies (Garner et al., 2014; Davis et al., 2015; Rammohan et al., 2015), we were able to observe about a 3.5-fold increase in the transcription product in the presence of CarD when compared to RNAP alone (Figure 3). To test the inhibitory activity of the peptides, we incubated *Mtb* RNAP holoenzyme with varying concentrations (10 μM – 1 mM) of the peptides (1–3) in the presence of CarD and initiated the transcription with the addition of the radiolabeled CTP. The densitometric analysis of *in vitro* transcription products suggested that peptide 2 showed the maximum inhibitory activity and was able to reduce transcription by approximately twofold at ∼50 μM concentration (Figures 3A,B). The calculated IC_50_ values of all the evaluated peptides are mentioned in Supplementary Table 2. In agreement with the computational studies, peptides 1 and 2 were most efficient in inhibiting CarD mediated transcription activation. However, in contrast to docking scores, peptide 2 showed higher *in vitro* CarD-mediated inhibitory activity. Based on our hypothesis, binding of the designed peptides will inhibit CarD/RNAP interactions, hence resulting in reduced CarD mediated activation. However, these peptides will not be able to inhibit the transcription completely as RNAP can perform basal transcription even in the absence of CarD.

## Discussion

In the current study, we report the rationally designed novel peptide-based inhibitors targeting the conserved PPI interface between the bacterial RpoBtr/transcription factor complexes. The target site investigated in our study has several advantages. Our results demonstrate that the target site is unique, does not share cross-resistance with the commercially available mycobacterial drugs, is highly conserved in both gram-positive and gram-negative bacteria, and no mutations were observed either in MDR or XDR strains of *Mtb*. Our *in silico* protein-peptide docking studies suggest that peptides 1 and 2 have a higher binding affinity for RpoBtr, thus highlighting their potential as the lead peptide-based RNAP β inhibitors. Our *in vitro* experiments suggest that the top ranked peptide 2 is highly stable, soluble in aqueous buffer, and capable of reducing CarD-mediated transcription activation.

Several studies have shown that peptides can be used as potential inhibitors to disrupt protein-protein interactions as they are highly selective, less toxic, and are effective against a broad range of targets (Wang et al., 2016). Hüsecken et al. (2013), designed the peptides mimicking σ^2.2^ which could be used for targeting *E. coli* RNAP σ^70^/core interface effectively (Hüsecken et al., 2013). Antisense peptide nucleic acids have been designed and demonstrated to inhibit the production of σ^70^ as an alternative therapeutic strategy against methicillin-resistant *Staphylococcus aureus* (Bai et al., 2012). Jeong et al. (2006), designed peptides mimicking *Mtb* RshA, an anti-σ factor, which could specifically inhibit the initiation of transcription by SigH, an alternative σ-factor *in vitro.* GE23077, a heptapeptide antibiotic target, binds to i and i + 1 site of the active center of RNAP (Ciciliato et al., 2004; Zhang et al., 2014). Microcin J25, a 21-mer peptide, binds near the secondary channel of RNAP and inhibits the transcription by competitively preventing the uptake of NTP (Mukhopadhyay et al., 2004). Besides several advantages offered by the peptide-based drugs, there are a few limitations (Otvos and Wade, 2014). However, the peptides designed in our study offer additional advantages as they were highly soluble in both water and aqueous buffers, and highly stable when stored over a long period of time at 4°C. We propose that conjugating the lead peptide 2 to antibodies, small molecules, or oligo ribonucleotides, incorporating non-native amino acids, cyclization, chemical stapling, or altering its chirality (Wang et al., 2016) may further improve its efficacy and selectivity. Our study suggests that targeting the newer site in the RpoB/transcription factor interface could be used as a potential site for developing novel drug molecules to tackle the increasing threat of MDR and XDR *Mtb* strains. Our study demonstrates the potential of using small molecules to target unexplored PPI sites on the transcription machinery of *Mtb* for possibly developing drugs for even MDR and XDR strains.

## Data Availability Statement

All datasets generated for this study are included in the article/Supplementary Material.

## Author Contributions

GK and KT proposed the concepts, designed the experiments, and wrote the manuscript. GK, SKap, and SKau carried out the experiments under the supervision of DD and KT. GK and KT analyzed and interpreted the results. All authors contributed to the article and approved the submitted version.

## Conflict of Interest

The authors declare that the research was conducted in the absence of any commercial or financial relationships that could be construed as a potential conflict of interest.

## Footnotes

1 http://www.rcsb.org

2 www.schrodinger.com
