# Supplementary material for: Structure-Guided Designing and Evaluation of Peptides Targeting Bacterial Transcription
Source: Front Bioeng Biotechnol. 2020 Sep 8;8:797. doi: 10.3389/fbioe.2020.00797 (PMC7505949; doi:10.3389/fbioe.2020.00797)
Supplement: Supplementary file 1 [file Data_Sheet_1.pdf]

Supplementary Information

Structure-guided designing and evaluation of peptides targeting bacterial transcription

Gundeep Kaur[a,b], Srajan Kapoor[a], Soni Kaundal[a,c], Dipak Dutta[d] and Krishan Gopal Thakur[a#]

[a]G N Ramachandran Protein Centre, Structural Biology Laboratory, Council of Scientific and Industrial Research-Institute of Microbial Technology (CSIR-IMTECH), Chandigarh, India
[b]Current Address: Department of Epigenetics and Molecular Carcinogenesis, University of Texas, MD Anderson cancer Center, Houston, Texas, USA
[c]Current Address: Verna and Marrs McLean Department of Biochemistry and Molecular Biology, Baylor College of Medicine, Houston, Texas, USA
[d]Molecular Microbiology Laboratory, Council of Scientific and Industrial Research-Institute of Microbial Technology (CSIR-IMTECH), Chandigarh, India

[#]**Address correspondence to:**
Krishan Gopal Thakur
Council of Scientific and Industrial Research-Institute of Microbial Technology,
Chandigarh-160036, India.
E-mail: krishang@imtech.res.in

Running Head:
Designing and validating peptide-based inhibitors for bacterial RNAP/transcription factor interface

Supplementary Figures

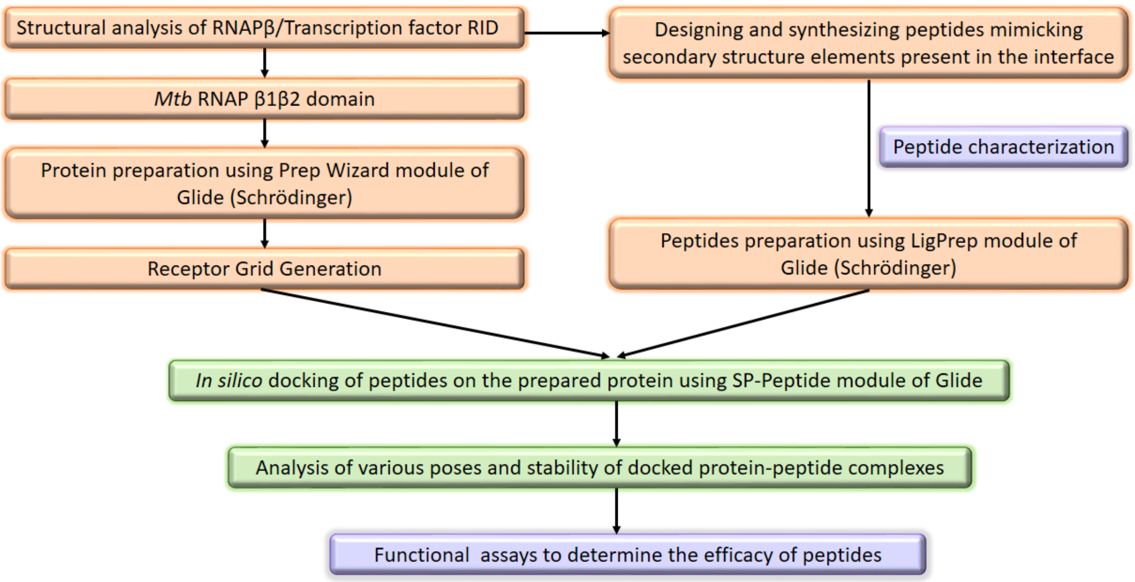

Supplementary Figure 1: The workflow used for structure based designing and evaluation of peptides capable of targeting bacterial transcription.

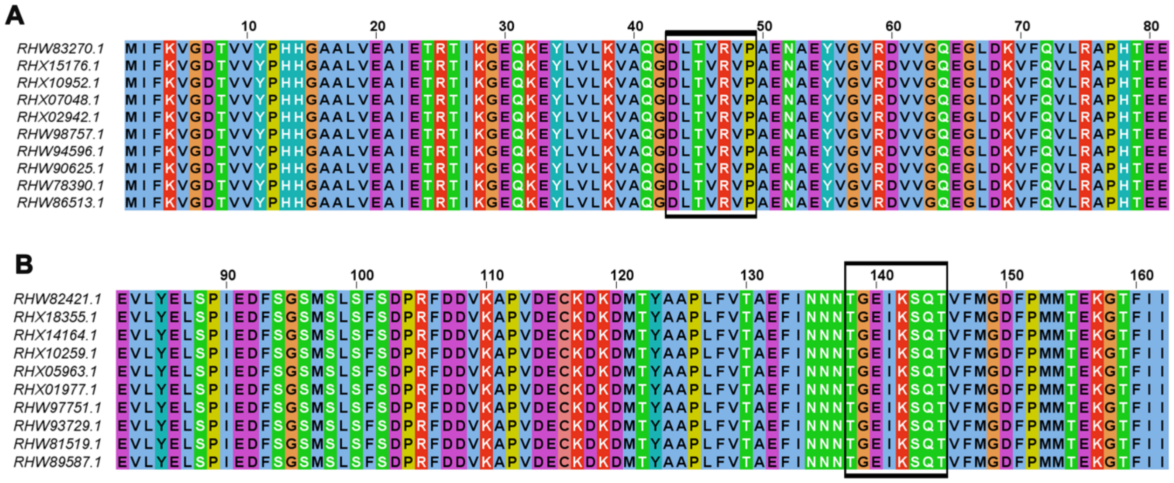

Supplementary Figure 2: The multiple sequence alignment of CarD (A) and RpoB (B) in MDR and XDR strains of *Mtb shows that* the residues involved in CarD/RpoB interaction (indicated in the black rectangular box) are highly conserved. The accession numbers of CarD and RpoB in MDR and XDR strains of clinical isolates are mentioned on the left.

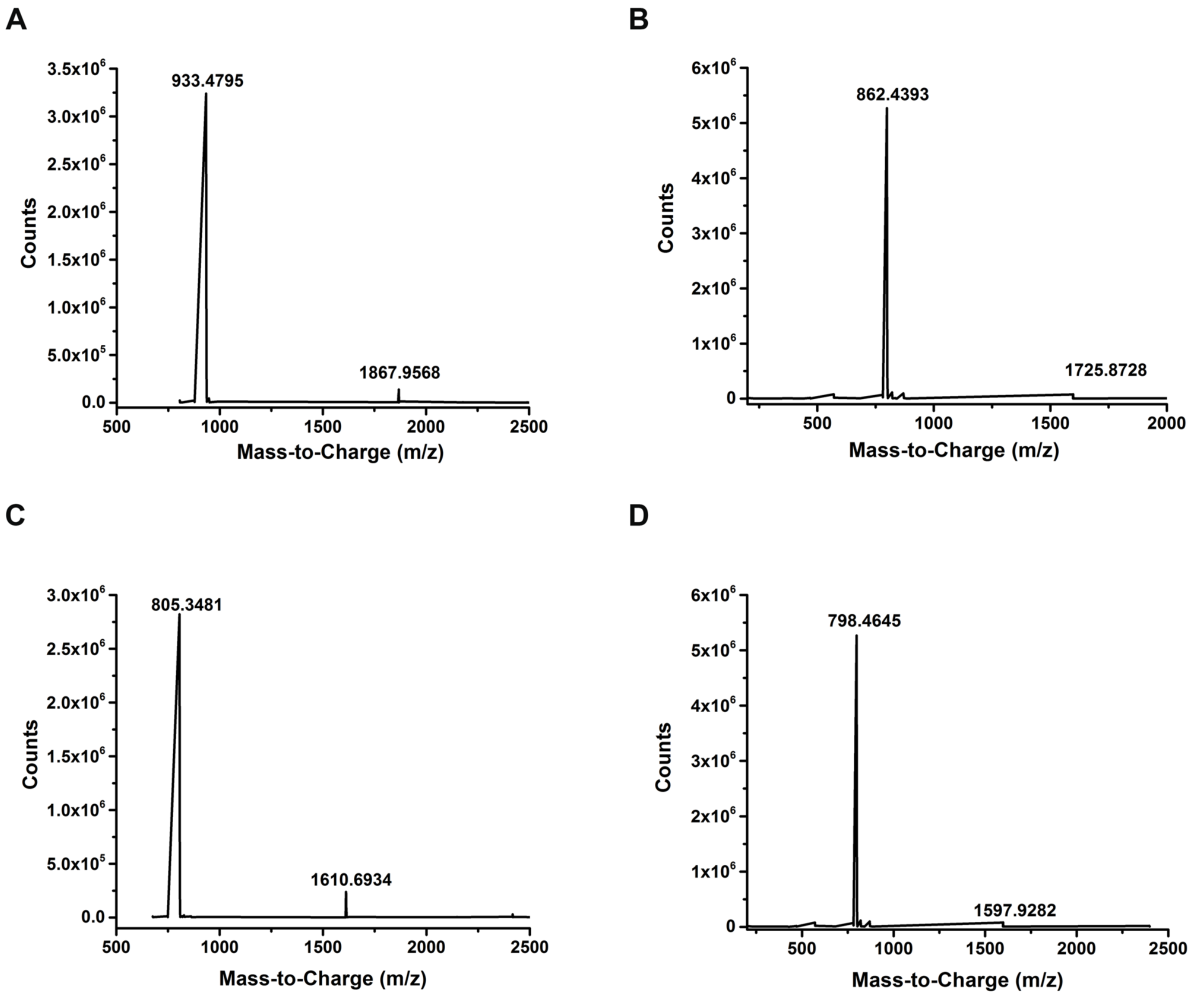

Supplementary Figure 3: The deconvoluted LC-MS spectra of all the peptides shows that the peptides were stable in water at 4 ºC when stored over a long period of time (A) The MW_{obs} of Peptide 1 is 933.48 Da (B) The MW_{obs} of Peptide 2 is 862.44 Da (C) The MW_{obs} of Peptide 3 is 798.47 Da (D) The MW_{obs} of Peptide 4 is 805.35 Da. The MW_{obs} matches with the MW_{exp} of all the peptides. $MW_{obs:}$ Observed Molecular weight and $MW_{exp:}$ Experimental Molecular weight.

A

B

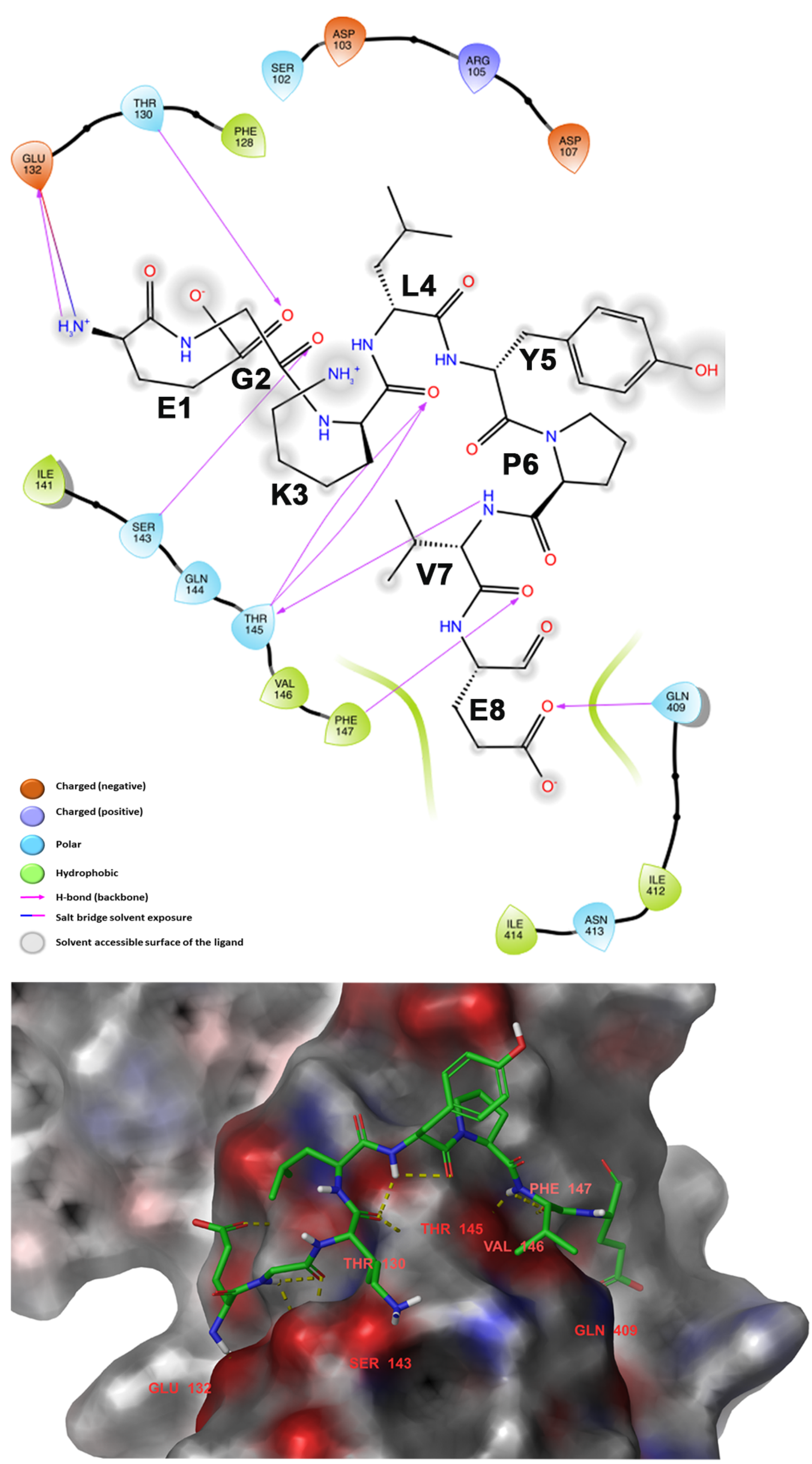

Supplementary Figure 4: The 2D representation (A), and surface representation (B), showing the binding mode and the key residues involved in the interactions between RpoBtr and the peptide1 identified in the molecular docking procedures.

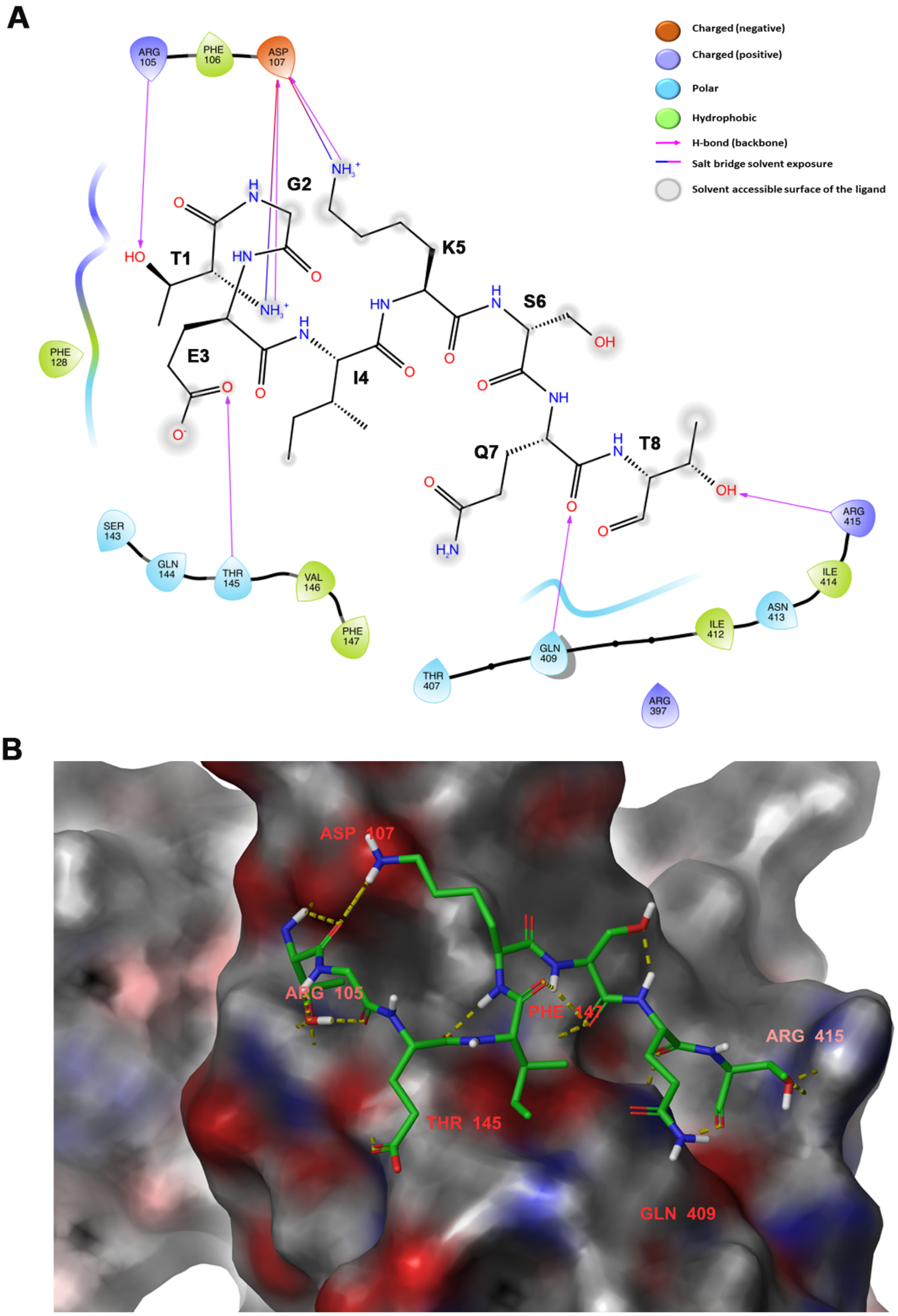

Supplementary Figure 5: The 2D representation (A), and surface representation (B), showing the binding mode and the key residues involved in the interactions between RpoBtr and the peptide 2 identified in the molecular docking procedures.

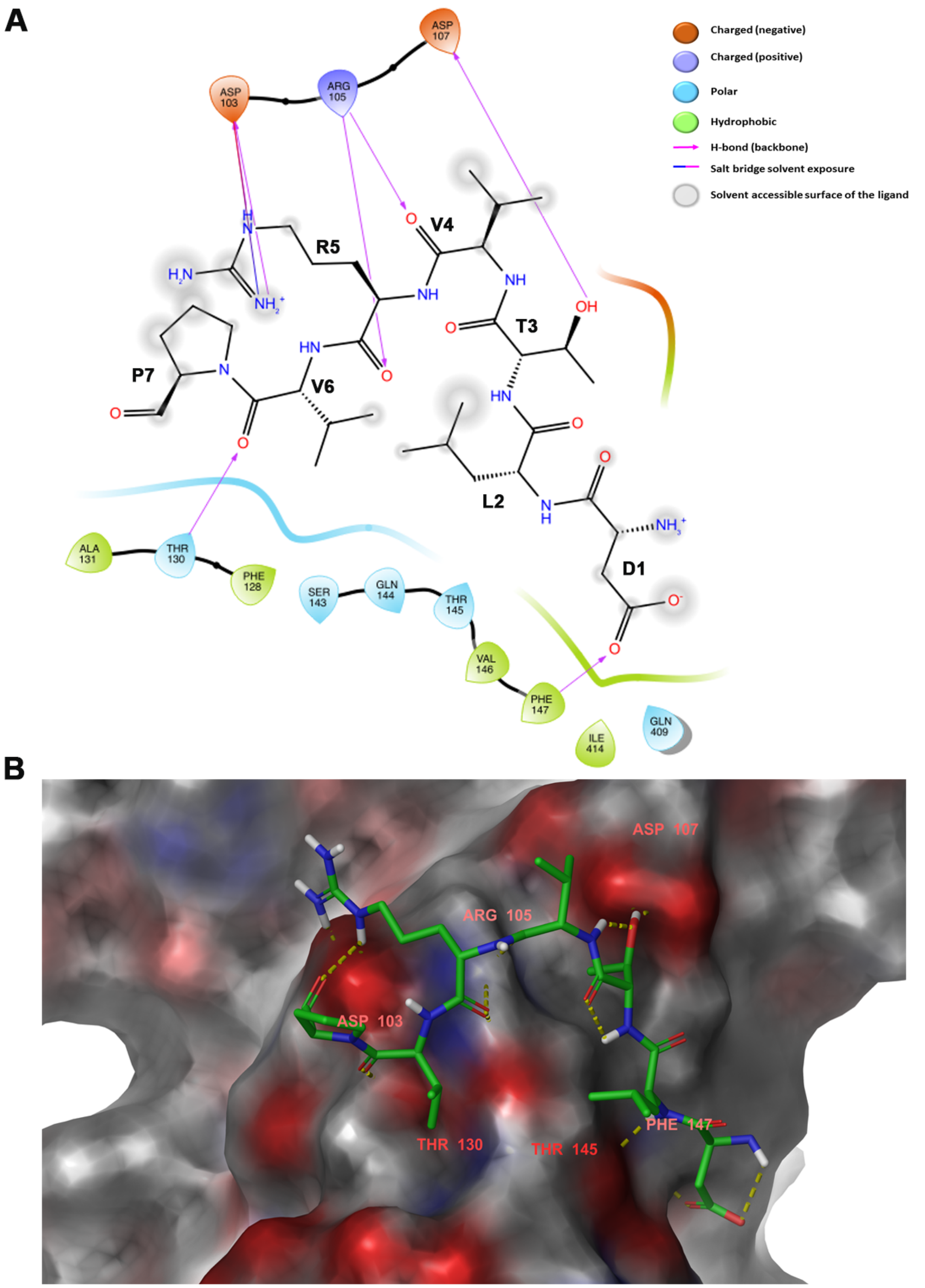

Supplementary Figure 6: The 2D representation (A), and surface representation (B), showing the binding mode and the key residues involved in the interactions between RpoBtr and the peptide 3 identified in the molecular docking procedures.

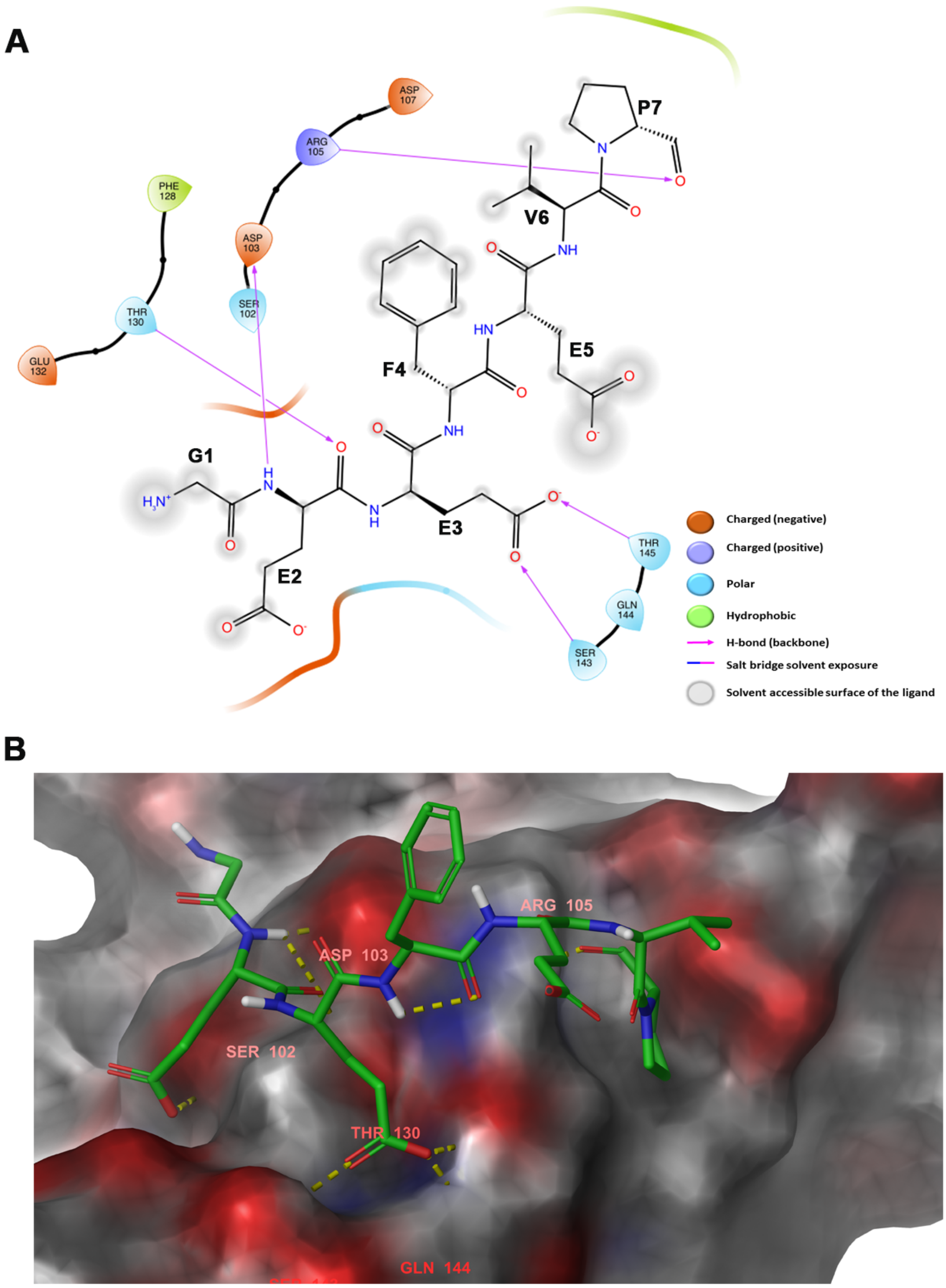

Supplementary Figure 7: The 2D representation (A), and surface representation (B), showing the binding mode and the key residues involved in the interactions between RpoBtr and the peptide 4 identified in the molecular docking procedures.

Supplementary Tables

Supplementary Table 1: Details of interface residues involved in PPI between conserved RNAP β/transcription factor complex.

Tth RNAP β1/TRCF-RID (PDB ID 3MLQ)		*Mtb* RpoBtr/CarD (PDB ID 4KBM)	
RNAP β1	TRCF-RID	RNAP β	CarD
T105	V343	T138	G42
G106	Y350	G139	D43
L107	G359	E140	L44
I108	K360	I141	T45
K109	L361	K142	V46
E110	Y362	S143	R47
	L363	Q144	V48
	P364	T 145	P49

Supplementary Table 2:

The calculated IC_{50} values of all the evaluated peptides used to inhibit *mycobacterial* transcription.

Peptide ID	IC_{50} (μM)
1	100
2	50
3	100

References:

1. **Micsonai A, Wien F, Kernya L, Lee YH, Goto Y, Refregiers M, Kardos J.** 2015. Accurate secondary structure prediction and fold recognition for circular dichroism spectroscopy. Proc Natl Acad Sci U S A **112:**E3095-3103.
